# Effects of Multikinase inhibitors on pressure overload-induced right ventricular remodelling

**DOI:** 10.1186/1476-9255-10-S1-P37

**Published:** 2013-08-14

**Authors:** Baktybek Kojonazarov, Akylbek Sydykov, Soni Savai Pullamsetti, Himal Luitel, Bhola K Dahal, Djuro Kosanovic, Xia Tian, Matthaeus Majewski, Christin Baumann, Steve Evans, Peter Phillips, David Fairman, Neil Davie, Chris Wayman, Iain Kilty, Norbert Weissmann, Friedrich Grimminger, Werner Seeger, Hossein Ardeschir Ghofrani, Ralph Theo Schermuly

**Affiliations:** 1 Universities of Giessen and Marburg Lung Centre (UGMLC), Giessen, Germany; 2 Department of Lung Development and Remodelling, Max-Planck Institute for Heart and Lung Research, Bad Nauheim, Germany; 3 Inflammation and Remodeling Research, Pfizer Cambridge Massachusetts, 02140, USA

## 

Little is known about the effects of current PAH therapies and receptor tyrosine kinase inhibitors on heart remodelling. We sought to investigate the effects of the multikinase inhibitors sunitinib (PDGFR-, VEGFR- and KIT-inhibitor) and sorafenib (raf1/b-, VEGFR-, PDGFR-inhibitor) on pressure overload induced right ventricular (RV) remodelling. We investigated the effects of the kinase inhibitors on hemodynamics and remodelling in rats subjected either to monocrotaline (MCT)-induced PH or to surgical pulmonary artery banding (PAB). MCT rats were treated from day 21 to 35 with either vehicle, sunitinib (1 mg/kg, 5 mg/kg and 10 mg/kg/day) or sorafenib (10 mg/kg/day). PAB rats were treated with vehicle, sunitinib (10 mg/kg/day) or sorafenib (10mg/kg/day) from day 7 to 21. RV function and remodelling were determined using echocardiography, invasive hemodynamic measurement and histomorphometry. Treatment with both sorafenib and sunitinib decreased right ventricular systolic pressure, pulmonary vascular remodelling, RV hypertrophy and fibrosis in MCT rats. This was associated with an improvement of RV function. Importantly, after PAB, both compounds reversed RV chamber and cellular hypertrophy, reduced RV interstitial and perivascular fibrosis, and improved RV function. We demonstrated that sunitinib and sorafenib reversed RV remodelling and significantly improved RV function measured via a range of invasive and non-invasive cardiopulmonary endpoints in experimental models of RV hypertrophy.

